# Reduction of Off-Flavor Generation in Soybean Homogenates: A Mathematical Model

**DOI:** 10.1111/j.1750-3841.2010.01733.x

**Published:** 2010-09

**Authors:** Nathan Mellor, Frances Bligh, Ian Chandler, Charlie Hodgman

**Affiliations:** Authors Mellor and Hodgman are with CPIB in the Multidisciplinary Centre for Integrative Biology, School of Biosciences, the Univ. of Nottingham, Sutton Bonington CampusLE12 5RD, UK. Authors Bligh and Chandler are with the Unilever Discover Colworth, Colworth Science ParkSharnbrook, Beds MK44 1LQ, UK

**Keywords:** lipoxygenase, model, n-hexanal, off-flavor, soybean

## Abstract

The generation of off-flavors in soybean homogenates such as n-hexanal via the lipoxygenase (LOX) pathway can be a problem in the processed food industry. Previous studies have examined the effect of using soybean varieties missing one or more of the 3 LOX isozymes on n-hexanal generation. A dynamic mathematical model of the soybean LOX pathway using ordinary differential equations was constructed using parameters estimated from existing data with the aim of predicting how n-hexanal generation could be reduced. Time-course simulations of LOX-null beans were run and compared with experimental results. Model L_2_, L_3_, and L_12_ beans were within the range relative to the wild type found experimentally, with L_13_ and L_23_ beans close to the experimental range. Model L_1_ beans produced much more n-hexanal relative to the wild type than those in experiments. Sensitivity analysis indicates that reducing the estimated K_m_ parameter for LOX isozyme 3 (L-3) would improve the fit between model predictions and experimental results found in the literature. The model also predicts that increasing L-3 or reducing L-2 levels within beans may reduce n-hexanal generation.

**Practical Application**: This work describes the use of mathematics to attempt to quantify the enzyme-catalyzed conversions of compounds in soybean homogenates into undesirable flavors, primarily from the compound n-hexanal. The effect of different soybean genotypes and enzyme kinetic constants was also studied, leading to recommendations on which combinations might minimize off-flavor levels and what further work might be carried out to substantiate these conclusions.

## Introduction

Soybean (*Glycine max)*is an important ingredient in many processed foods. However, their use may be limited by the generation, following homogenization, of so-called off-flavors (Lei and Boatright 2001; Lozano and others 2007). The presence of these flavors or aromas may cause variability in product quality, particularly beverages made using crushed soybeans. It is believed that in many plants the off-flavors are generated by a range of aldehydes known as C6-aldehydes or green leaf volatiles (GLVs) (Matsui 2006; Pulvera and others 2006). These GLVs are generated by the action of enzymes in the lipoxygenase (LOX) pathway (Feussner and Wasternack 2002). Of particular interest for food manufacturers is the means of controlling one such GLV, n-hexanal.

LOX is ubiquitous in plants and catalyses the dioxygenation of polyunsaturated fatty acids (PUFAs) containing (1Z,4Z)-pentadiene systems (Brash 1999; Liavonchanka and Feussner 2006). Soybean seeds are rich in 2 such suitable substrates, linoleic acid (LA) and α-linolenic acid (Liu 1997). Before beans are crushed, LOX and LA are separated within the cell, but following homogenization are mixed and begin to react to form the products of the LOX pathway, notably n-hexanal. The products of the LOX reaction are the 13- and 9-hydroperoxides of the PUFA substrate (Brash 1999). Soybean seeds have 3 LOX isozymes (L-1, L-2, and L-3), which vary in product positional specificity and kinetic behavior (Axelrod and others 1981). If LA is the substrate, then certain specific 13-hydroperoxide isomers formed go on to be cleaved by hydroperoxide lyase, a CYP74, to form n-hexanal and 12-oxo-(Z)-9-dodecenoic acid (Matoba and others 1985b; Pulvera and others 2006). The 2 LOX product isomers that go on to form n-hexanal, 13HOD-S(Z,E), and 13HOD-R(Z,E) (see nomenclature), are formed in different proportions, as well as at different rates, by the 3 LOX isozymes (Andre and Funk 1986; Fukushige and others 2005). At higher pH, n-hexanal may itself be converted to n-hexanol by alcohol dehydrogenase (ADH), which has less impact on flavor quality (Matoba and others 1989).

There have been several studies using LOX-null strains of soybean, which aim to elucidate the mechanism by which n-hexanal and other GLVs are formed (Matoba and others 1985a, 1985b, 1989; Pulvera and others 2006). In particular, studies using various existing soybean strains that lack one or more LOX isozymes show how different soybean genotypes produce different concentrations of n-hexanal following homogenization (Takamura and others 1991; Zhuang and others 1991; Nishiba and others 1995). From some of these studies, kinetic parameters can be derived that can be used to model the biological system mathematically.

While many laboratory studies for soybean n-hexanal generation have been published, no mathematical model of the LOX pathway was found that uses the existing data to try and describe observed behavior. This work has created a mathematical model of the soybean LOX pathway at near-neutral pH that produces time-course predictions of n-hexanal concentration comparable with existing laboratory data. Also the model has been used to predict and compare the effect of removing one or more LOX isozymes from the model. Further analysis may then suggest possible methods to reduce the generation of n-hexanal following soybean homogenization, or experiments that will help determine the source of off-flavor generation.

## Materials and Methods

### Kinetic parameters

**Figure 6:**
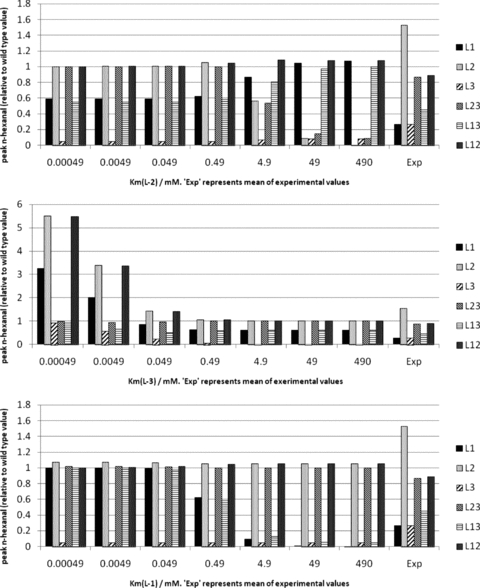
Peak n-hexanal values relative to “wild type” for each type of simulated null bean for increasing K_m_ parameter values for L-1, L-2, and L-3 isoenzymes. The final set of values marked “Exp” represent the mean experimental values found in the literature (Table 3).

Bild and others (1977) gives values for K_m_ and V_max_ for purified L-1 at both pH 6.8 and pH 9.0. Unfortunately, explicit values and K_m_ or V_max_ under similar conditions for L-2 or L-3 were not found in the literature. Takamura and others (1991) gave measurements at pH 6.5 of relative LOX activity for wild-type beans containing all 3 isozymes, and for 3 lines of mutant beans, each only possessing just one of the isozymes. By assuming that the ratios of these results correspond to the relative V_max_ of the 3 LOX isozymes, estimates of the different V_max_ parameters were made. The same K_m_ parameters were used for L-2 and L-3 as for L-1. The significance of the selection of K_m_ values was later tested by sensitivity analysis (Figure 6). The parameters used in the model for the 3 LOX reactions are given in Table 1.

**Table 1:** Michaelis–Menten kinetic parameter estimates used for the 3 LOX isozymes and HPL in the model. Estimates calculated from Bild and others (1977), Takamura and others (1991), and Matoba and others (1985b).

Model enzyme	K_m_/mmol mL^−1^	V_max_/μmol mL^−1^min^−1^mg protein	V_max_/μmol mL^−1^min (15 mg protein mL^−1^)
L-1	0.49	0.55	8.25
L-2	0.49	2.6	39
L-3	0.49	0.17	2.55
HPL (13HOD-R(Z,E))	0.05	19	285
HPL (13HOD-S(Z,E))	0.05	2.6	38.5

**Table 3:** Comparison of model null bean simulation (100 minutes) results with experimental data. All figures are n-hexanal concentrations relative to the wild type value given.

Genotype	Model	Zhuang and others (1991)	Matoba and others (1985a)	Matoba and others (1985b)	Nishiba and others (1995)	Takamura and others (1991)	Pulvera and others (2006)
L_123_	1.00	1.00	1.00	1.00	1.00	1.00	1.00
L_1_	0.80	0.50	n/a	0.18	0.49	0.11	0.06
L_2_	1.06	0.60	1.73	0.94	2.51	1.39	2.00
L_3_	0.07	n/a	n/a	n/a	0.40	0.10	0.29
L_12_	1.04	0.89	1.17	0.59	n/a	n/a	n/a
L_13_	0.70	0.63	0.33	0.38	n/a	n/a	n/a
L_23_	1.00	0.73	0.90	0.97	n/a	n/a	n/a
L_0_	0.00	n/a	n/a	n/a	0.22	0.10	0.06

The relative activity of Hydroperoxide Lyase (HPL) against the different LA hydroperoxide isomers is given by Matoba and others (1985b). This is given relative to the value of 100% for 13HOD-S(Z,E), with the only other isomer found to have significant activity as substrate for HPL being 13HOD-R(Z,E) with a mean relative activity of 13.5%. The V_max_ parameter used of 19 μmol·mg/min protein for 13HOD-S(Z,E) was the mean value given for wild-type beans. V_max_ for 13HOD-R(Z,E) was then estimated as 13.5% of this value. A K_m_ value of 40 to 60 μmol is given, and the mid value of 50 μmol has been used in the model for both active substrates.

### Initial concentrations

For simplicity, the initial concentration of all metabolites in the model, except the active enzymes and the initial LA substrate, is set to zero. An estimate of initial LA concentration of 67 nmol/mL has been used. This is based on data for the fatty acid composition of typical soybeans given by Liu (1997) and scaled relative to the total protein concentration given by Matoba and others (1985a) of 10 to 20 mg protein/mL. To account for the difference in V_max_ for purified enzyme and for whole bean homogenate, the assumption was made that in wild-type beans L-1, L-2, L-3 are present in similar quantities (Takamura and others 1991) and that each constitutes approximately 1% of total protein (Brash 1999). The final set of V_max_ and K_m_ estimates is given in Table 1.

### Product ratios

Data for the ratio of 13- and 9-HOD isomers produced by each of the LOX isozymes is taken from Andre and Funk (1986) for the L-1 isozymes, and from Fukushige and others (2005) for L-2 and L-3. For L-1, product ratios are given for pH 7 and are assumed to approximate values for pH 6.8, while values for L-2 and L-3 are given at pH 6.8. The product ratios for each LOX isozyme used in the model are shown in Table 2.

**Table 2:** Ratios of product isomers in the model for each LOX isozyme at pH 6.8 and pH 9.0. Data from Andre and Funk (1986) and Fukushige and others (2005).

Product	L-1 (% of total product)	L-2 (% of total product)	L-3 (% of total product)
13HOD-S(Z,E)	57.4	75.1	6.8
13HOD-R(Z,E)	14.4	2.3	5.9
13HOD-S(E,E)	5.0	2.5	13.6
13HOD-R(E,E)	1.2	1.5	10.7
9HOD-S(Z,E)	16.2	12.7	21.8
9HOD-R(Z,E)	4.0	2.6	21.8
9HOD-S(E,E)	1.4	1.8	9.8
9HOD-R(E,E)	0.4	1.6	9.7

### Modeling methods

**Figure 1:**
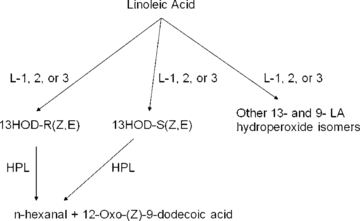
Metabolic pathway modeled using ordinary differential equations in Copasi.

The availability of K_m_ and V_max_ kinetic parameters for L-1 and HPL (Bild and others 1977, Matoba and others 1985b), and the relative activity of the 3 LOX isozymes (Takamura and others 1991), leads to the use of a mathematical model with Michaelis–Menten kinetics to simulate the biochemical network of the LOX pathway. Copasi (http://www.copasi.org) is a desktop software application, free for noncommercial use, designed for the modeling of such biochemical networks, and was used to create a deterministic model using a set of ordinary differential equations. Time-course data of the concentrations of the different metabolites in the model could then be generated for further analysis, for different parameter sets, and null-bean simulations. The biochemical network modeled is shown in Figure 1.

### Assumptions

For the model, the 1st assumption is that the activity of all 3 LOX isozymes is governed by Michaelis–Menten kinetics, and that once the product is disassociated from the LOX enzyme, the reaction is irreversible. Second, there is an assumption that each of the 8 possible 13- or 9-hydroperoxide isomers is produced at a different, fixed ratio for each of the 3 LOX isozymes. Also assumed is that oxygen is freely available for the LOX reaction, and is not rate limiting. For the HPL reaction, Michaelis–Menten kinetics were also used. It is assumed that HPL is present in similar quantities, has similar substrate specificity, and kinetic parameters, for all soybeans, regardless of which LOX enzymes are present. Only one substrate, free LA, is present in the model. Therefore, there is an assumption that n-hexanal is only produced via the LOX pathway, and that only free LA is a suitable substrate. The final initial assumption is that there are no feedback or inhibitory effects from any of the products or by-products generated by the model over the time course.

To model the predicted behavior of wild-type beans that contain all 3 LOX isozymes, the combined action of the 3 separate reactions is assumed to have an additive effect. Each reaction L-1, L-2, and L-3 competes for the LA substrate, and each produces the same range of 8 possible products in different proportions. The transient concentrations of these 8 products are the sums of those produced by all 3 reactions. In addition, by setting V_max_ for one or more LOX isozymes in the model to zero, the effect of using real beans lacking one of more LOX isozymes can be simulated.

The 2nd step in the model pathway is the cleaving of 13HOD-S(Z,E) or 13HOD-R(Z,E) by HPL to form n-hexanal and 12-oxo-(Z)-9-dodecenoic acid using the parameters given by Matoba and others (1985b). Model simulations were run to give time-course data for the generation of n-hexanal for all the different combinations of LOX-null beans.

The model is (will be) available for download in SBML format at http://www.biomodels.net.

## Results and Discussion

### Primary LOX products

**Figure 2:**
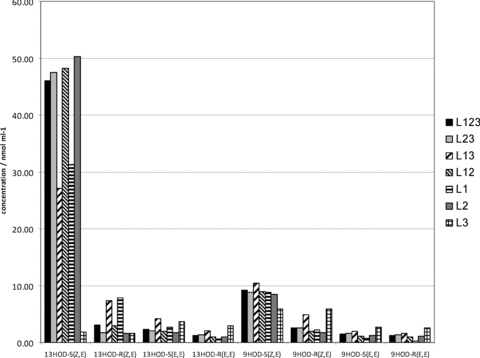
LOX products by type after 100 min model time for different simulated null beans.

Figure 2 shows the concentration of products after 100 min predicted by the combined LOX model without the HPL reactions included. Also shown is the predicted concentration of the primary LOX products after 100 min for each of the possible combinations of LOX isozymes. In each case, with the exception of simulated L-3 only beans (L_3_, see nomenclature), the majority of the product formed is 13HOD-S(Z,E). The products formed in greatest concentrations by the simulated L_3_ beans are 9HOD-S(Z,E) and 9HOD-R(Z,E). The preferred substrate of HPL, 13HOD-S(Z,E), was modeled in the next step of the LOX pathway, and so the relative concentrations in which it is formed in different bean genotypes are significant. In the model, the L_2_ beans formed the most 13HOD-S(Z,E), closely followed by the L_12_, L_23_, and L_123_(wild type) beans, then at around one-third lower concentrations the L_1_ beans, the L_13_ beans, and finally the L_3_ beans producing by far the lowest concentration.

### Null-bean simulation compared with experimental data

Several sets of experimental results are available in the literature that gives n-hexanal generation for different LOX-null beans under various different experimental conditions. Table 3 summarizes the results by showing the n-hexanal generated by each LOX-null bean relative to the wild type for the different experimental results, alongside the simulated values.

Model L_2_, L_3_, and L_12_ values all fell within the range of values obtained experimentally. L_13_ and L_23_ beans were also fairly close to the range of experimental values with 0.70 and 1.00 model compared to experimentally derived ranges of 0.38 to 0.63 and 0.73 to 0.97, respectively. The L_1_ beans show the greatest difference between the model and the experimental data, with a model value of 0.80 times the wild-type n-hexanal concentration compared with a much lower range of 0.06 to 0.50 times the wild type in the lab experiments. Where values for L_0_ beans were given in experimental data, there was a background level of n-hexanal generation observed of 0.06 to 0.22 times the wild-type value.

Qualitatively at least, the differences between simulated null beans were mostly fairly similar to those observed in the null-bean experiments. In the literature, the soybean genotype that consistently produces the most n-hexanal is that which only contains the L-2 isozyme (L_2_) (Takamura and others 1991; Nishiba and others 1995; Pulvera and others 2006). This is also true of the model. This is to be expected as n-hexanal is derived mainly from 13HOD-S(Z,E) that is produced in highest proportions by L-2. Since there is a finite amount of substrate, when other LOX isozymes are present more of it is converted into other products that are not converted into n-hexanal. Similarly, it is to be expected that L_3_ beans produce relatively low amounts of n-hexanal, as L-3 generates the least 13HOD-S(Z,E) of the 3 isozymes. In the model, the L_3_ beans do produce the least n-hexanal, and within the range concentrations relative to the wild type found in the literature. Experimentally, however, while L_3_ beans produce very low amounts of n-hexanal, the L_1_ beans also produce similarly low or lower amounts (Matoba and others 1985b; Takamura and others 1991; Nishiba and others 1995; Pulvera and others 2006). This is not true of the model results, in which L_1_ beans produce more than 7 times the amount of n-hexanal as the L_3_ beans. The simulated beans in which just one isozyme is missing produce n-hexanal in a similar order to that found in most experiments surveyed, with L_12_ producing the most, followed by L_23_, wild-type beans, and finally L_13_ producing the least (Matoba and others 1985a; Zhuang and others 1991).

It is unclear why beans containing just the L-1 isozyme should produce similarly low amounts of n-hexanal as the beans containing just L-3. Takamura and others (1991) measures LOX activity for L_1_ beans as more than 3 times that for L_3_ beans at pH 6.5, and the proportion of 13HOD-S(Z,E) is given as 57.4% for L-1 (Andre and Funk 1986) compared to just 6.8% for L-3 (Fukushige and others 2005). Other possibilities for low n-hexanal generation by L_1_ beans is that other enzymes affecting generation are absent or present in different quantities, or that there is less free fatty acid substrate present. Matoba and others (1985) does show that there is some variation in fatty acid composition between bean genotypes, but there is no data for L-1 only beans. It may also be that the kinetics of the reactions does not proceed as assumed in the model and that some feedback or inhibitory effects of the by-products generated may occur.

### Time-course results compared with experimental data

**Figure 3:**
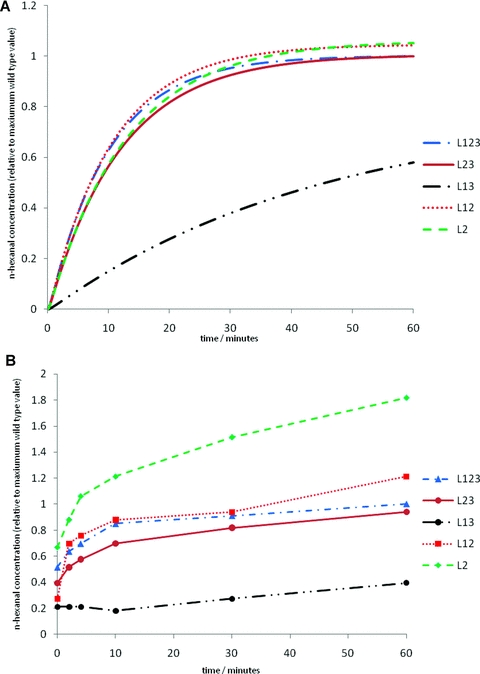
Comparison of model n-hexanal concentration for (A) various simulated null beans and (B) experimentally obtained results (Matoba and others 1985a), in which no standard errors were given.

Using the estimate of initial free LA of 67 nmol/mL, estimated using the value of 10 to 20 μmol/mL protein content given by Matoba and others (1985a) results in a model prediction of around 50 nmol/mL n-hexanal produced by wild-type beans. The value given in the paper is around 0.3 nmol/mL for wild-type Suzuyakata beans, more than a 10-fold decrease. This may be due to the degradation of free LA by other pathways, or an overestimation of initial LA or LOX concentrations. In order to compare the relative n-hexanal generation for different bean types between model and experimental data, the data were therefore plotted relative to the maximum wild-type value for both model predictions and experimental data, respectively. Figure 3A shows the comparison between different simulated LOX-null beans, and Figure 3B shows the experimental results (taken directly from the paper itself, which gives no standard errors and a pH range of 6.5 to 7.0). Comparing the overall pattern of the results, some correlation is observed. The L_13_ (L-2 null) beans show clearly the lowest generation of n-hexanal in both model and experiment, with 0.4 and 0.6 times the peak wild-type value, respectively. The experimental data then show (in order of increasing peak n-hexanal) the L_23_, the L_123_ (wild type), and the L_12_ beans steadily increasing from an initial value to a peak after 60 min of around 0.8 (L_23_) and 1.2 (L_12_) times the peak wild-type value. The model predicts a similar order, with L_12_ beans producing slightly more n-hexanal than the wild type, and L_23_ slightly less, although the difference between the 3 curves appears smaller. There are 2 main qualitative differences between the experimental and model datasets. The 1st is the curves representing the L_2_ beans. Experimentally, these beans consistently produced the most n-hexanal throughout the time course, reaching a maximum value of around 1.8 times the wild-type value after 60 min. In contrast, in the model, while the peak value for L_2_ is still the highest predicted, it is much closer to the values predicted for the L_12_, wild type, and L_13_ beans, and for much of the time course, the predicted n-hexanal is lower than that of other beans. The experimental data for all beans show an initial base level of n-hexanal that is not accounted for in the model. For simplicity, initial n-hexanal concentration is set to zero in the model, but the experimental data give a mean initial concentration of around 0.14 times peak wild-type n-hexanal or 0.4 nmol/mL. In addition, Table 3 shows that in experiments where beans with none of the LOX isozymes present (L_0_), n-hexanal is still generated in concentrations comparable to the null beans L_1_ and L_3_. It is possible that this is a steady state value of n-hexanal concentration prior to homogenization, when LA and LOX are isolated. After homogenization, LA and LOX are mixed, with the end result that n-hexanal concentration increases. Alternatively, there may be another metabolic pathway by which n-hexanal is generated.

**Figure 4:**
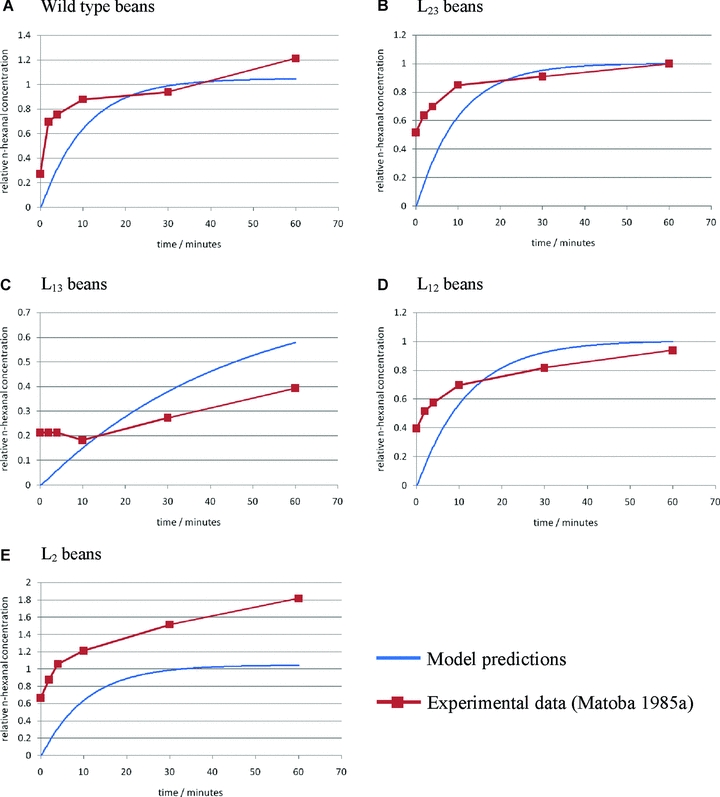
Comparison by individual null-bean type between model predictions and experimental results (Matoba and others 1985a).

Figure 4 shows the same data but with a direct comparison between model and experimental values for each type of bean. Again there appears to be a rough correlation, with the exception of the L_2_ beans, and the presence of initial concentrations of n-hexanal observed experimentally in all bean types. In the present model, 13HOD-S(Z,E) and 13HOD-R(Z,E) concentrations are only depleted by the activity of HPL. In reality, these molecules may by degraded or metabolized by other pathways, and the rates of these pathways will have an effect on n-hexanal concentration. An experiment in which 13HOD-S(Z,E) and 13HOD-R(Z,E) levels are recorded while HPL activity is blocked or otherwise inhibited would enable an estimation of these rates, and enable their inclusion in the model. If we assume that these parameters and those relating to HPL remain the same regardless of the presence or absence of the 3 LOX isozymes, then while varying them will have an effect on the absolute concentrations of n-hexanal, they should have little or no effect on the relative concentrations obtained for the different null bean simulations.

The model is based on data at or near-neutral pH. Some data are however available at higher pH. In particular, it is shown that purified L-1 enzyme has peak activity at around pH 9.0 to 9.5 (Axelrod and others 1981), and as may be expected that LOX activity for L_1_ beans is also much higher at pH 9.5 (Takamura and others 1991). Andre and Funk (1986) do give data for the relative concentrations of product isomers produced by L-1 at pH 9, but the precise values for each isomer is not given explicitly, and no statistical errors are given. While Fukushige and others (2005) give excellent data for isomer ratios for L-2 and L-3 at pH 6.8, no data are given on how the ratios differ at higher pH. A consistent set of experiments in which product ratios for all 3 isozymes are determined at a range of pHs would be useful in extending the model to simulate behavior at different pH. The behavior of HPL and estimates for the K_m_ of the LOX isozymes at increasing pH would also be required to extend the model in this way. Matoba and others (1989) suggests a means by which n-hexanal is converted to n-hexanol at higher pH by the activity of ADH. In this experiment, n-hexanal peaked after around 15 min after homogenization before returning to near its initial value. Since n-hexanol has been reported as having a less negative effect on soybean flavor, increasing this conversion of n-hexanal to n-hexanol may be desirable.

### Sensitivity analysis

**Figure 5:**
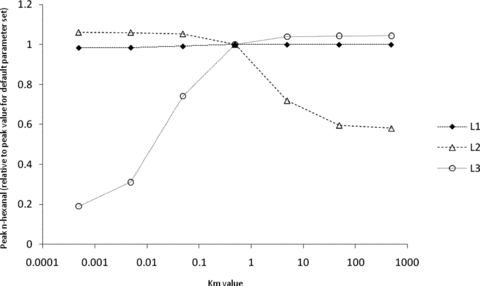
Simulated peak n-hexanal concentration against varying K_m_ parameter values for L-1, L-2, and L-3 isoenzymes in “wild type” beans. Peak n-hexanal is given relative to the value at default K_m_ (0.49 mM).

Changing the kinetic parameters of each of the 3 LOX isozymes in the model affects the peak n-hexanal concentration for simulated null beans to varying degrees (Figure 5). Most of the variation was seen by reducing L-3 K_m_ (increasing V_max_), with peak n-hexanal of around one-fifth that predicted using the default value following a 1000-fold decrease. Increasing L-3 K_m_ 1000-fold only results in a very slightly higher peak n-hexanal concentration. Increasing L-2 K_m_ (reducing V_max_) 1000-fold reduces peak n-hexanal concentration to around 0.6 times the value for the default parameter, while decreasing 1000-fold has relatively little effect. Increasing or decreasing L-1 K_m_ (decreasing or increasing V_max_) has the least effect relatively of the 3 isozymes, with peak n-hexanal remaining in a similar range for all the parameter values tested. Reducing or decreasing V_max_ values of the 3 LOX isozymes in the model should have a similar effect as changing individual LOX protein concentration and so more precise data for the concentration of the different LOX isozymes in the different bean types would be useful in testing the model further. The analysis would suggest that increasing levels of L-3 or reducing L-2 would have the greatest effect on reducing n-hexanal generation.

Clearly, altering the parameter estimates used for the LOX reactions will affect model predictions for n-hexanal generation. Figure 6 shows how different parameter values will affect the comparison between “wild type” and each of the simulated null beans, and how these predictions compare with experimentally obtained values. The set of experimental values (marked “Exp”) represents the mean of the values in Table 3 sourced from the literature. The pattern of experimental results appears to be high generation of n-hexanal for L_2_ beans (around 1.5 time wild type), followed by L_23_ and L_12_ beans (both around 0.9 times wild type), then L_13_ (around 0.5 times wild type), with the lowest n-hexanal generation in the L_1_ and L_3_ beans (both less than 0.3 times wild type). When comparing to the default model values (K_m_= 0.49 mM), the major differences are that values are much closer to the wild-type beans for both L_2_ and L_1_ beans. L_23_, L_13_, and L_12_ simulated beans all generate slightly more n-hexanal than observed experimentally, while simulated L_3_ generates less than the mean experimental value.

The best fit between model and experiment in this set of results is given by decreasing L-2 K_m_ by a factor of 10. This increases the L_2_ n-hexanal near to the experimental value of around 1.5 times wild type, while increasing the L_3_ n-hexanal nearer to the experimental value. However, this also has the effect of increasing the value for L_1_ beans nearer to the L_23_ beans, and the L_12_ bean n-hexanal increases along with the L_2_ beans, so the fit is still not perfect. Increasing L-1 K_m_ slightly may improve the model by reducing n-hexanal for the L_1_ and L_13_ beans toward the experimental values, while leaving values for the other beans unaffected. Reducing L-1 K_m_ from the default value of 0.49 mM does not improve model performance as it increases the value for both the L_1_ and L_13_ beans closer to the wild-type value, away from the experimental values. Increasing L-2 K_m_ reduces the fit between the model and experimental values, as it simultaneously increases L_1_ and reduces L_2_ n-hexanal. Reducing L-2 K_m_ has little effect on relative levels between the different beans. More sophisticated parameter fitting and optimization may be required in order to find the best fit between the available experimental results and model predictions.

## Conclusion

While the model does partially reflect the behavior observed experimentally, there is some room for improvement. In particular, the model predicts that increasing L-3 or reducing L-2 levels within beans may reduce n-hexanal generation, as may be expected. On the other hand, the difference between the behavior of simulated and laboratory L_1_ beans is not accounted for. Sensitivity analysis for LOX K_m_ values suggests that parameter sets that compare more favorably with experimental results could be found by parameter fitting or optimization techniques. The results of Matoba and others (1989) suggest that including the effects of increasing pH and the inclusion of the reaction converting n-hexanal to n-hexanol by ADH at higher pH will be vital in using the model to predict optimum conditions for reducing n-hexanal generation during soybean processing.

## Nomenclature

L subscript 1, 2, or 3 refers to the LOX isozymes present in that bean (for example, L_13_ contains LOX isozymes L-1 and L-2)

13HOD-S(Z,E), 13S-hydroperoxy-(9Z,11E)-octadecadienoic acid

13HOD-R(Z,E), 13R-hydroperoxy-(9Z,11E)-octadecadienoic acid
